# Brexpiprazole in patients with schizophrenia with or without substance use disorder: an observational study

**DOI:** 10.3389/fpsyt.2023.1321233

**Published:** 2023-12-04

**Authors:** Ginevra Lombardozzi, Giada Trovini, Emanuela Amici, Georgios D. Kotzalidis, Filippo Perrini, Valeria Giovanetti, Alessandro Di Giovanni, Sergio De Filippis

**Affiliations:** ^1^Villa Von Siebenthal Neuropsychiatric Hospital and Clinic, Genzano di Roma, Italy; ^2^NESMOS Department, Faculty of Medicine and Psychology, Sant'Andrea Hospital, Sapienza University, Rome, Italy; ^3^Department of Neuroscience, Section of Psychiatry, Fondazione Policlinico Universitario Agostino Gemelli IRCSS, Rome, Italy; ^4^Centro Lucio Bini, Rome, Italy; ^5^ASL Roma 6, Rome, Italy

**Keywords:** antipsychotic medications, brexpiprazole, partial dopamine D_2_ receptor agonists, schizophrenia, substance use disorder

## Abstract

**Background:**

Partial dopamine D_2_ receptor agonists are used for psychotic symptoms in adults with schizophrenia spectrum disorders. Recently, interest surged for partial dopamine D_2_ receptor agonists in substance use disorders (SUDs). Since it is believed that SUDs decrease the efficacy of pharmacotherapy of underlying psychiatric disorders, we tested the efficacy of the partial D_2_ agonist brexpiprazole in patients with schizophrenia who were either comorbid with a SUD (SUD group) or not comorbid (non-SUD) to assess treatment response and the effect of brexpiprazole on substance craving in SUD.

**Methods:**

We included patients with DSM-5/DSM-5-TR schizophrenia (using SCID-5-CV) aged 18–66 years with either comorbid SUD or non-SUD to treat with brexpiprazole 4 mg/day for 6 months during February–October 2022. Patients were assessed with the Clinical Global Impressions–Severity (CGI-S) scale, the 24-item Brief Psychiatric Rating Scale (BPRS), and the Positive And Negative Syndrome Scale (PANSS) at baseline, weekly for the first 2 months and monthly for the next four. Furthermore, we assessed substance craving in SUD with a visual analog scale for craving (VAScrav) at the same timepoints.

**Results:**

The total sample was 86 (85 analysable) 18- to 64-year-old (mean 39.32 ± 14.09) patients with schizophrenia [51 men (59.3%) and 35 women (40.7%)], of whom 48 SUD (55.8%) (37 men and 11 women) and 38 non-SUD (44.2%) (14 men and 24 women). No serious or persistent adverse events developed over the study period, but one patient dropped out for subjective akathisia. Results indicated the main effects of time with improvements over the course of the study for CGI-S, BPRS, and PANSS in both SUD and non-SUD groups and the entire sample, and for VAScrav in SUD. Brexpiprazole was associated with similar significant improvements in both groups at the 6 month endpoint compared to baseline.

**Conclusion:**

Treatment with brexpiprazole for 6 months improved psychotic symptoms in patients with schizophrenia, independently from whether they belonged to the SUD or the non-SUD group; hence, SUD comorbidity did not confer treatment resistance to brexpiprazole. Furthermore, in the SUD group, we observed reduced substance craving.

## 1 Introduction

Schizophrenia is a severe mental disorder with a pooled annual incidence of 15 per 100,000 people (1). It is a chronic condition that has huge health, social, and economic impacts on patients, their families and caregivers, and on the entire society; it ranked 20th among the leading causes of disability in 2019 (1). Its treatment is still unsatisfactory and is the focus of debate, with newly proposed drugs based on recent developments in the pathophysiology of the disorder (2–4) going beyond the classical dopaminergic hypothesis.

A few months passed since the first report of environmental indifference induced by chlorpromazine (5), which prompted Henri Laborit to advise Delay and Deniker (6) to use the drug in psychiatric patients and to report the efficacy of chlorpromazine in purported schizophrenia cases (7) and in manic agitation (8). From that time until the first rudimentary draft of the dopaminergic theory (9), 11 years elapsed, and a further 3 years were required until its accomplished formulation (10). In the meantime, phenothiazines were considered antihistaminics that failed to induce adequate analgesia, and all antipsychotic drugs produced in these years were variations of methylene blue and chlorpromazine structures. The discovery of the butyrophenone haloperidol also followed a serendipitous path, with the young chemist Bert K. F. Hermans synthesizing the drug on 11 February 1958 at Janssen (11), while Paul A.J. Janssen's group was playing around with the structure of pethidine in an attempt to discover stronger pain killers (12); the discoverers published their results 1 year later (13), but had already passed the molecule to Divry et al. (14, 15) and Paquay et al. (16), who performed the first encouraging clinical trials, something that would not have been feasible in current years for ethical reasons. At these times, the dopamine receptor was unknown to the scientific community, and dopamine was considered to be only a noradrenaline precursor. Carlsson et al. (17, 18) identified dopamine as an independent transmitter in the brain in the late 1950s. Seven more years were still needed to formulate a form of the dopaminergic hypothesis for schizophrenia that posed hyperdopaminergia as the pathological basis of schizophrenia (10). The theory underwent various modifications, with additions, specifications, and enrichment concerning other transmitters and modulators, such as glutamate, γ-aminobutyric acid (GABA), serotonin (5-hydroxytryptamine, 5-HT), acetylcholine, endorphins, other peptides, and adenosine, but the idea that increased mesolimbic dopaminergic activity linked to the development of schizophrenia was never disproved and all adjunctive mechanisms had to fit this idea, as drugs without mesolimbic antidopaminergic (direct or indirect) activity are ineffective. It was hypothesized that abnormally developing dopaminergic projections to the prefrontal cortex affected GABAergic and glutamatergic transmissions in the dorsolateral prefrontal cortex (DLPFC) and its feedback to the mesolimbic dopaminergic system (19). Realizing that dopaminergic activity in the prefrontal cerebral cortex was hypoactive and in the mesolimbic system was hyperactive led to the development of drugs that could slow the activity of dopamine in the mesolimbic system, as all neuroleptics and antipsychotics do, but enhance it in the DLPFC (20), which is the seat of executive functions that help individuals to deal with the tasks of everyday life. These drugs are able, through partial agonist activity on the D_2_ group of receptors (D_2_, D_3_, and D_4_), to increase dopaminergic activity in the DLPFC and by increasing the dose to block dopaminergic receptors in the mesolimbic system. Simultaneously, they block 5-HT_2A_ (and 5-HT_6_ and 5-HT_7_) receptors (21) and partially activate 5-HT_1A_ receptors (22). Hence, the properties of the “atypical” or “second generation” antipsychotics apply to these molecules. This group of drugs comprises aripiprazole, cariprazine, and brexpiprazole, which have shown comparable effectiveness in schizophrenia studies, although each maintains its own unique profile (23). In particular, brexpiprazole is more potent at the 5-HT_7_ receptors than the other two drugs (Supplementary Table 1). Inhibiting the 5-HT_7_ receptor has been associated with positive cognitive effects (24, 25), which are essential for recovery from schizophrenia (26).

Currently, there are more than 20 pharmacotherapeutic options to treat schizophrenia symptoms (27, 28) and some focus on non-dopaminergic mechanisms (29), although these mechanisms ultimately affect dopaminergic transmission (30). When choosing a prescription, clinicians should balance efficacy with safety and adverse events. The latter may impair the patient's quality of life (QoL) and lead to treatment discontinuation (31, 32). This, in turn, may be followed by symptom exacerbation, which is the main reason for subsequent hospitalization (33). Hospitalization, besides constituting a traumatic event in a psychiatric patient's life (34), is also related to increasing healthcare costs and social burdens (35).

Schizophrenia treatment is arduous even after treating the acute phase. The main task is to treat the acute phase but also to prevent relapses and lead the patient to recovery, thus ensuring socialization and reintegration into the community. Antipsychotics often need to be taken for very long periods, thus increasing the probability of adverse events, which prompt patients to discontinue medication and decrease adherence (36). A meta-analysis of clinical trials compared 32 commonly prescribed oral antipsychotics and found similar efficacy rates, while the greater differences regarded adverse events (37). This meta-analysis found weak effect sizes for brexpiprazole compared to clozapine but also confirmed a low potential for adverse events. The D_2_ dopamine receptor partial agonist antipsychotics, due to their potential to increase prefrontal cortical dopamine release, which is related to motivation and cognition (38–40), can decrease the symptoms of mood disorders, such as some core symptoms [but not all (41)] of major depressive disorder (MDD) or bipolar depression. In fact, this group of antipsychotics is used, both in monotherapy and in augmentation, in the treatment of mood (41) and personality disorders (42). While the FDA accepted some antipsychotic drugs as monotherapy in bipolar depression, it does not recommend any antipsychotic alone for unipolar depression.

Brexpiprazole (7-[4-[4-(1-benzothiophen-4-yl)piperazin-1-yl]butoxy]quinolin-2(1H)-one) was first approved in the US by the FDA in 2015 for schizophrenia in adults and pediatric patients older than 13 years, and as an add-on to an antidepressant drug for MDD in adults (43). In 2023, its indication was expanded to the treatment of agitation associated with dementia due to Alzheimer's disease (44). In Europe, it is indicated for schizophrenia in adults (45).

Brexpiprazole was found to be suitable for long-term adult schizophrenia treatment, as it shows a favorable adverse event profile, thus ensuring safety, besides reducing both positive and negative symptoms; this way, it achieves the goals of increasing patient's socialization and reintegration into the community (46).

Brexpiprazole displays a high affinity for serotonin, dopamine, and noradrenaline receptors. It strongly binds (*K*_i_ < 1 nM), 5HT_1A_ and 5HT_2A_ serotonin receptors, D_2_ dopamine receptors, and α_1B_ and α_2C_ adrenoceptors (47, 48). It is a partial agonist at 5HT_1A_ serotonin and D_2_ dopamine receptors and an antagonist at 5-HT_2A_ serotonin receptors and α_1_ and α_2_ adrenoceptors (47, 48). Brexpiprazole shows a fairly high affinity (*K*_i_ < 5 nM) for D_3_ dopamine-, 5HT_2B_, 5HT_7_ serotonin-, and α_1A_ and α_1D_ adrenergic receptors, a moderate affinity (*K*_i_ = 19 nM) for H_1_ histamine receptors, and low affinity (*K*_i_ > 1,000 nM) for M_1_ muscarinic cholinergic receptors (47, 48). Compared to aripiprazole and cariprazine, brexpiprazole binds the D_2_ dopamine and the 5-HT_2A_ serotonin receptors strongly and displays more powerful partial agonist activities on 5-HT_1A_ and 5-HT_2C_ serotonin receptors. Due to its lower intrinsic activity at D_2_ receptors and higher binding affinities for 5-HT_1A/2A_ receptors than aripiprazole, brexpiprazole would have a favorable antipsychotic potential without D_2_ receptor agonist- and antagonist-related adverse effects (48). Furthermore, due to its greater 5-HT_7_-blocking ability compared to other dopamine D_2_ partial agonists [Supplementary Table 1; (49–51)], brexpiprazole has more robust effects on the cognitive impairment associated with schizophrenia (24, 25, 48).

Brexpiprazole and aripiprazole have low propensities to induce extrapyramidal symptoms (EPS). However, the low EPS risk of brexpiprazole is more likely dependent on its agonist properties on presynaptic 5-HT_1A_ receptors, while that of aripiprazole is less sensitive to 5-HT_1A_ receptor antagonism, as shown in a preclinical study (52).

Both antipsychotics reduce the symptoms of schizophrenia, but brexpiprazole seems to show a peculiar reduction in impulsivity; this latter should lead to better tolerability with a lower incidence of akathisia (53). Brexpiprazole administered to patients with schizophrenia and impulsivity was associated with decreased right ventrolateral prefrontal cortex (VLPFC) activation and decreased stop-signal reaction time (SSRT), supporting a benefit of brexpiprazole on inhibition-related brain activation and behavior (54).

The blockade of mesolimbic receptor D_2_ results in the inhibition of the reward and reinforcement circuits. While dopamine D_2_ receptor antagonism reduces positive psychotic symptoms in schizophrenia, it may worsen negative symptoms such as apathy, avolition, reduced motivation, and anhedonia (55). This partly explains an increase in tobacco and substance use in patients treated with classical antipsychotic drugs (56, 57). Substance use disorders (SUDs) are highly comorbid with psychotic disorders (58). Patients with psychosis appear to be particularly vulnerable to the consumption of psychoactive drugs (59). Negative symptoms, either primary or fostered by antipsychotics, may promote the use of psychostimulant drugs, and the latter may be used to treat negative symptoms (60). However, psychostimulant drugs are not easy to manage and cannot be used for long periods of time.

Dose-dependent reductions of cocaine self-administration in rhesus monkeys were obtained when drugs with 5-HT_2C_ receptor agonist and 5-HT_1A_ receptor partial agonist properties were administered (61). 5-HT_2A_ antagonist activity and partial agonist activity on 5-HT_1A_ and 5-HT_2C_ receptors could be active in reducing stimulant drug consumption in patients with SUDs comorbid with psychotic disorders. Thus, in the light of its receptor-binding profile, brexpiprazole appears to be a valid treatment for psychosis and a particularly suitable drug for patients with psychotic symptoms and concomitant SUD.

There are perspectives for the new class of dopamine receptor partial agonists in various psychiatric disorders and neurological diseases, and many molecules are now being tested (62, 63). It appears that those directed to the D1 group of dopamine receptors (D_1_/D_5_), such as tavapadon, will not work in schizophrenia, but will be effective in Parkinson's disease (64), while psychotic disorders will respond to the partial agonism of the D_2_ group of receptors. Recently, there has been a suggestion that by focusing on the partial agonism of this group, especially D_3_, we could develop drugs that may prove useful in SUDs (65–68). Such drugs can reduce the psychotic symptomatology of schizophrenia in patients with SUD (65). We employed the D_2_ dopamine receptor partial agonist, brexpiprazole, to assess its efficacy in patients with schizophrenia with or without a comorbid SUD in a study with an open-label design. In this study, we do not report on safety in detail, which was however assessed and will be the object of a future study. Our intent was to assess the efficacy of brexpiprazole in reducing psychotic symptoms in both subpopulations. We also aimed to evaluate if the presence of a comorbid SUD conferred treatment resistance and to assess the effect of brexpiprazole on the craving for the substance used in that specific SUD for 6 months.

## 2 Materials and methods

### 2.1 Patients

We conducted an observational study on inpatients with a diagnosis of schizophrenia hospitalized at the Villa Von Siebenthal neuropsychiatric hospital. Recruitment began on 1st February 2022 and ended on 31st October 2022.

Patients aged from 18 to 66 years were eligible if they had (1) a diagnosis of DSM-5/DSM-5-TR schizophrenia and SUD (cannabis, synthetic cannabinoids, cocaine, amphetamines, opioids, ketamine/phencyclidine or other NMDA receptor inhibitors, khat and other alkaloid cathinones, and alcohol or polysubstance use disorder) or (2) schizophrenia without SUD (69, 70). We admitted SUD patients who were receiving their specific SUD pharmacological treatment, such as methadone, buprenorphine, and naltrexone, or benzodiazepines and gabapentinoids. All patients were initially inpatients, discharged after 1 month and followed-up as outpatients thereafter.

Exclusion criteria were the presence of a comorbid major psychiatric disorder other than schizophrenia; high risk of suicide as assessed with the Columbia-Suicide Severity Risk Scale (C-SSRS) (71); comorbidity with severe organic diseases (autoimmune or systemic connective tissue diseases, treatment-resistant hypertension, type 1 diabetes, metabolic syndrome, severe cardiovascular diseases, and major neurological diseases); history of epilepsy, head injury, electroencephalographic (EEG) abnormalities, and neurodevelopmental disorders; intelligence quotient (IQ) < 75, as assessed with the Wechsler Adult Intelligence Scale (WAIS) (72); unwillingness to participate, and inability to sign the informed consent for oneself or, in case of inability, unwillingness/refusal of the legal guardian to sign.

After meeting the inclusion criteria and not meeting the exclusion criteria, patients were explained study aims and methods and provided free, informed consent. The study received approval from the local ethical committee (CE Lazio 2, Rome, Italy; protocol number 331-306-00387). It was conducted in accordance with the Principles of Human Rights, as adopted by the World Medical Association at the 18th WMA General Assembly, Helsinki, Finland, June 1964, subsequently amended by the 64th WMA General Assembly, held in Fortaleza, Ceará, Brazil, in October 2013.

### 2.2 Treatment

If the included patients were antipsychotic drug naïve or antipsychotic drug-free for at least 2 weeks, they were immediately treated with brexpiprazole, following the recommended titration from 1 mg once daily to adjustment to 2–4 mg once daily. If they were on other antipsychotic medications, they were prescribed brexpiprazole after a proper wash-out of at least 2 weeks. Once reaching the appropriate dose for each patient (based on clinical course and clinician's decision), usually the target dose of 4 mg/day in monotherapy, the regimen was maintained for 6 months. Patients were not allowed to take other antipsychotic drugs or antidepressants throughout the study period; the only medications allowed were those specifically used for each SUD, i.e., methadone, buprenorphine and naltrexone, and benzodiazepines and gabapentinoids for anxiety and insomnia.

### 2.3 Study assessments

We followed up with our patients for 6 months, evaluating their psychopathology with psychometric scales.

Schizophrenia and SUD (cannabis, synthetic cannabinoids, cocaine, amphetamines, opioids, ketamine/phencyclidine or other inhibitors of NMDA receptors, khat and other cathinone alkaloids, and alcohol and polysubstance use disorder) were diagnosed by professional psychiatrists using SCID-5-CV (73); eligibility was based on schizophrenia diagnosis. Patients were regularly tested for drug use both at intake and during the study.

Patients were assessed at baseline, every week for 2 months, and every month for a further 4 month period (study endpoint at 6 month follow-up) with the following instruments.

To rate psychopathology, we used the Clinical Global Impressions–Severity scale (CGI-S) (74), the 24-item Brief Psychiatric Rating Scale (BPRS) (75), the Italian version (76), and the Positive And Negative Syndrome Scale (PANSS) (77). To evaluate craving in patients with SUD, we used the visual analog scale for craving (VAScrav) (78). The latter rates craving from 0 (no craving) to 10 (the most intense craving according to patient's experience).

The BPRS has been developed from a previous 18-item version (79), which has been factorialised in the following five subscales: anxiety-depression, anergy, thought disorders, activity, and hostility (80). A similar factor structure has also been obtained for the expanded 24-item version (81), so we decided to maintain this five-factor solution as the best fit, although factoralisations of the BPRS have been very inconsistent (82, 83). Our primary goal was to assess the efficacy of brexpiprazole through PANSS, CGI-S, and BPRS scores.

Patients affected by SUD were meant to be compared to those without SUD (non-SUD) to assess if comorbid SUD could hinder brexpiprazole psychosis treatment and if the same treatment is associated with changes in substance craving. Adverse events were recorded as reported.

### 2.4 Statistical analysis

Frequency distributions and descriptive statistics were performed to analyse the sample. We used Student's *t*-test for analyzing point differences between the two samples in continuous variables, with all two-tailed analyses, univariate analysis of variance (ANOVA) for analyzing course differences of continuous variables and the chi-squared test (χ^2^) for nominal variables after ensuring normal distribution with the Shapiro and Wilk test (84) and sphericity with the Mauchly W-test (85). Data were analyzed using the IBM Statistical Package for the Social Sciences (SPSS) Version 23 (IBM, Armonk, New York, 2016). Significance was set at *p* < 0.05.

## 3 Results

**Figure 1:**
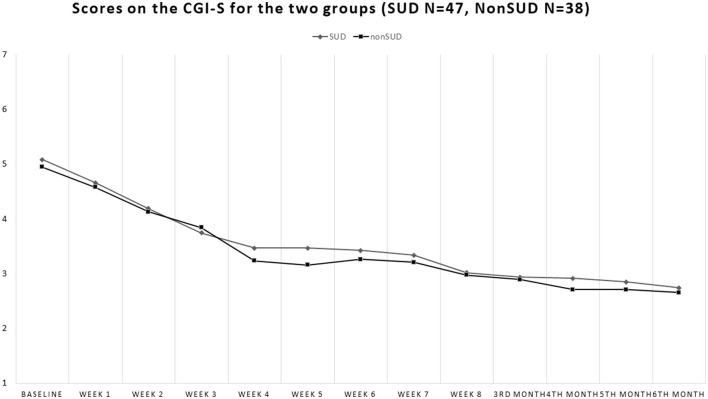
Drop of Clinical Global Impressions (CGI-S) scores during the study in the groups with comorbid substance use disorder (SUD) and the non-comorbid group (non-SUD). The two groups look quite similar in their scores on this scale.

**Figure 2:**
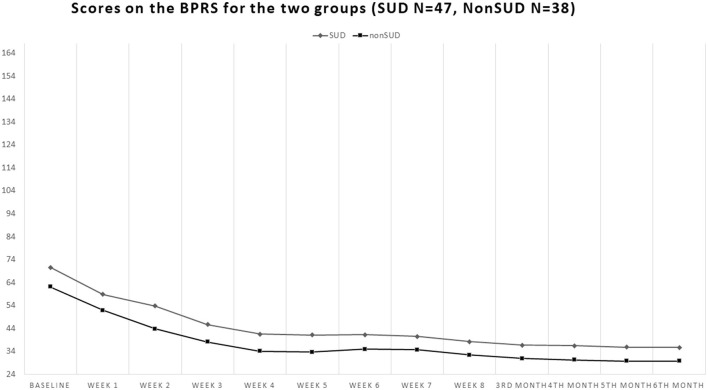
Course of the scores on the 24-item Brief Psychiatric Rating Scale-Expanded version (BPRS-24) in the groups with comorbid substance use disorder (SUD) and in the non-comorbid group (non-SUD). Both groups obtained about a 50% reduction from baseline to endpoint, but the non-SUD group scored constantly lower throughout the study, indicating that its participants fared better than the comorbid SUD group.

**Figure 3:**
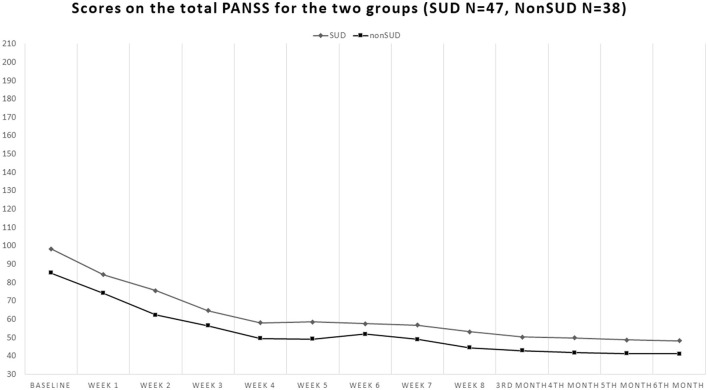
Course of the total scores of the Positive And Negative Syndrome Scale (PANSS) in the groups with comorbid substance use disorder (SUD) and the non-comorbid group (non-SUD). Both groups obtained >50% reduction from baseline to endpoint, compatible with clinical response, but similarly to what occurred with the BPRS-24, the non-SUD group scored constantly lower throughout the study, indicating that the non-comorbid group was clinically better than the comorbid SUD group.

**Figure 4:**
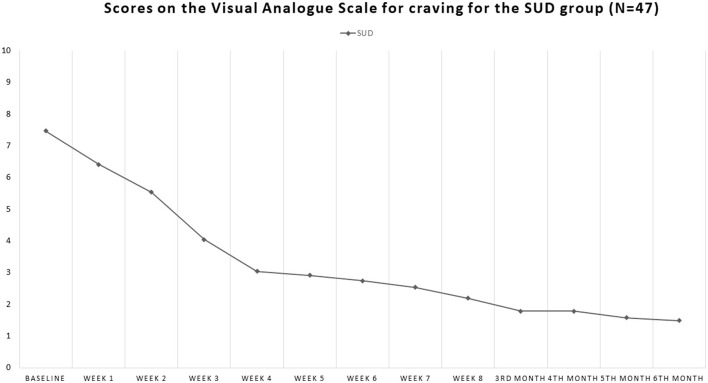
Course of the scores on the Visual Analog Scale for craving (VAScrav) in the SUD comorbid group. This scale is rated by the patient from 0 to 10; at baseline, it was 7.47 and by the 6 month endpoint had dropped to 1.49, i.e., by >80%.

Our sample consisted of 86 patients with schizophrenia, with 51 men (59.3%) and 35 women (40.7%). Of these, 48 patients had comorbid SUD (55.8%), 37 men and 11 women, whereas 38 did not have a substance use disorder in comorbidity (non-SUD) (44.2%), 14 men and 24 women. Patients' ages ranged from 18 to 64 years (mean 39.32, standard deviation SD = 14.09). The sociodemographic characteristics of the sample, along with the SUD types, are shown in Table 1. The patients' scores on the clinical scales are shown in Figures 1–**4**. Of the 86 patients who were included in the sample, 85 were analyzed because one woman of the non-SUD group requested to withdraw after 1 week on 1 mg brexpiprazole due to subjectively perceived akathisia.

**Table 1:** Sociodemographic characteristics of the sample with schizophrenia.

	**Study sample (*n* = 86)**	**Men (*n* = 51; 59.30%)**	**Women (*n* = 35; 40.70%)**	**Test**	***P***
Age in years (x ± SD) *t*-test	39.51 ± 14.53	36.12 ± 11.72	44.46 ± 16.49	−2.71	**0.008**
**Marital status**, ***N*** **(%) [**χ^2^ **test]**					
Single	60 (69.77%)	43 (84.32%)	17 (48.57%)	12.77	**0.047**
Married	14 (16.28%)	5 (9.80%)	9 (25.71%)		
Separated/divorced	11 (12.79%)	3 (5.88%)	8 (22.86%)		
Widowed	1 (1.16%)	0 (0%)	1 (2.86%)		
**Educational level**, ***N*** **(%) [**χ^2^ **test]**					
Primary school	2 (2.33%)	0 (0%)	2 (5.71%)	11.83	0.066
Middle school	34 (39.53%)	19 (37.26%)	15 (42.86%)		
High school	42 (48.84%)	28 (54.90%)	14 (40%)		
College/University, Master classes, Specialty, Ph.D.	8 (9.30%)	4 (7.84 %)	4 (11.43%)		
**Presence of alcohol (AUD) or Substance Use Disorder (SUD)**, ***N*** **(%) [**χ^2^ **test]**					
No AUD or SUD	38 (44.19%)	14 (27.45%)	24 (68.57%)	14.23	**0.0002**
AUD and/or SUD	48 (55.81%)	37 (72.55%)	11 (31.43%)		
° Polysubstance	24 (50%)	17 (45.95%)	7 (63.64%)	3.35	0.763
° Cannabis	14 (29.16%)	13 (35.14%)	1 (9.09%)		
° Cocaine	5 (10.42%)	4 (10.81%)	1 (9.09%)		
° Alcohol	5 (10.42%)	3 (8.10%)	2 (18.18%)		

Significant differences in bold characters. AUD, alcohol use disorder; N, number; SD, standard deviation; SUD, substance use disorder; t-test; Student's t-test; x, mean; χ^2^, chi-square test.

At baseline, the non-SUD group scored 4.95 ± 0.61 on the CGI-S, while the SUD group scored 5.06 ± 0.84 [Student's *t* = 0.71; *p* = 0.48, not significant (ns)], and at endpoint, they scored 2.66 ± 0.48 and 2.74 ± 0.67, respectively (Student's *t* = 0.67; *p* = 0.51, ns). Both groups showed significant decrements from baseline to endpoint Student's *t* = 18.12; *p* < 0.00001 for the non-SUD group and Student's *t* = 15.01; *p* < 0.00001 for the SUD group (Figure 1).

At baseline, the non-SUD group scored 62.08 ± 13.81 on the BPRS (total score), while the SUD group scored 70.55 ± 19.02 (Student's *t* = −2.30; *p* = 0.024, with the latter scoring significantly higher), and at endpoint, they scored 6.82 ± 1.43 and 35.64 ± 12.92, respectively (Student's *t* = −2.61; *p* = 0.01, with the SUD group scoring even more significantly higher than the non-SUD). Both groups showed significant decreases from baseline to endpoint Student's *t* = 13.25; *p* < 0.00001 for the non-SUD group and Student's *t* = 10.41; *p* < 0.00001 for the SUD group (Figure 2).

Regarding BPRS subscales, the non-SUD group scored 18.76 ± 4.31 at baseline on the BPRS anxiety/depression subscale, while the SUD group scored 16.55 ± 4.15 (Student's *t* = 2.40; *p* = 0.019, with the latter scoring significantly lower), and at endpoint, they scored 7.50 ± 2.60 and 7.26 ± 3.04, respectively (Student's *t* = 0.39; *p* = 0.69, ns). Both groups showed similar significant decreases from baseline to endpoint Student's *t* = 13.80; *p* < 0.00001 for the non-SUD group and Student's *t* = 12.39; *p* < 0.00001 for the SUD. On the anergy subscale of the BPRS, the non-SUD group scored 14.92 ± 5.28 at baseline and the SUD group scored 15.00 ± 4.52 (Student's *t* = −0.07; *p* = 0.94, ns), while at endpoint, they scored 6.58 ± 2.11 and 7.62 ± 2.91, respectively (Student's *t* = 1.84; *p* = 0.07, ns). Both groups showed similar significant decreases from baseline to endpoint (Student's *t* = 9.04; *p* < 0.00001 for the non-SUD group and Student's *t* = 9.42; *p* < 0.00001 for the SUD). On the thought disorder subscale of the BPRS, the non-SUD group scored 13.29 ± 6.19 at baseline, while the SUD group scored 18.45 ± 8.06 (Student's *t* = −3.25; *p* = 0.0017, with the SUD group scoring significantly higher), and at endpoint, they scored 6.58 ± 2.11 and 9.19 ± 4.31, respectively (Student's *t* = −3.01; *p* = 0.004, with the SUD group scoring higher and the gap between the two remaining). However, both groups showed similar significant score decreases from baseline to endpoint (Student's *t* = 6.29; *p* < 0.00001 for the non-SUD group and Student's *t* = 6.95; *p* < 0.00001 for the SUD). On the activity subscale of the BPRS, the non-SUD group scored 10.37 ± 4.00 at baseline and the SUD group 12.72 ± 5.75 (Student's *t* = −2.14; *p* = 0.035; the SUD group scored significantly higher), while at endpoint, they scored 5.76 ± 1.17 and 7.02 ± 2.51, respectively (Student's *t* = −2.85; *p* = 0.006, with the SUD group scoring higher and the gap between the two remaining and even enlarging). In any case, both groups showed similar significant score decreases from baseline to endpoint (Student's *t* = 6.81; *p* < 0.00001 for the non-SUD group and Student's *t* = 6.23; *p* < 0.00001 for the SUD). On the hostility/suspiciousness subscale of the BPRS, the non-SUD group scored 4.61 ± 2.63 at baseline and the SUD group scored 7.77 ± 4.26 (Student's *t* = −4.00; *p* = 0.0001, with the SUD group scoring significantly higher), while at endpoint they scored 3.11 ± 0.39 and 4.46 ± 2.27, respectively (Student's *t* = −3.54; *p* = 0.0007, with the SUD group scoring higher and the gap between the two tending to close but remaining). At any rate, both groups showed similar significant score decreases from baseline to endpoint (Student's *t* = 3.48; *p* = 0.0008 for the non-SUD group and Student's *t* = 4.74; *p* < 0.00001 for the SUD).

On the PANSS, the non-SUD group obtained a baseline total score of 85.29 ± 14.94 and the SUD 98.40 ± 21.83 (Student's *t* = −3.15; *p* = 0.002, with SUD scoring higher). At endpoint, they obtained scores of 41.08 ± 10.03 and 48.17 ± 16.69 (Student's *t* = −2.30; *p* = 0.024, with the SUD group still scoring higher, but with the gap tending to close). Both groups showed significant large decreases from baseline to endpoint (Student's *t* = 15.15; *p* < 0.00001 for the non-SUD group and Student's *t* = 12.53; *p* < 0.00001 for the SUD) (Figure 3).

Concerning PANSS subscales, baseline scores on the positive PANSS subscale were 12.95 ± 5.59 for the non-SUD group and 19.62 ± 9.94 for the SUD group (Student's *t* = −3.69; *p* = 0.0004, with SUD scoring higher), while at endpoint they were 7.87 ± 1.54 and 9.66 ± 3.81, respectively (Student's *t* = −2.72; *p* = 0.008, with SUD continuing to score higher). In both groups, reductions in PANSS positive scores were significant Student's *t* = 5.39; *p* < 0.00001 for the non-SUD and Student's *t* = 6.41; *p* < 0.00001 for the SUD group. Baseline scores on the negative PANSS subscale were 22.00 ± 6.86 for the non-SUD and 24.26 ± 5.74 for the SUD group (Student's *t* = −1.65; *p* = 0.103, ns), while at endpoint, they scored 9.79 ± 3.60 and 11.57 ± 4.48, respectively (Student's *t* = −1.99; *p* < 0.05, with the SUD group scoring marginally higher than the non-SUD). Both groups showed significant score decreases from baseline to endpoint (Student's *t* = 9.72; *p* < 0.00001 for the non-SUD group and Student's *t* = 11.95; *p* < 0.00001 for the SUD). On the general psychopathology PANSS subscale, baseline scores were 50.39 ± 10.68 for the non-SUD and 54.62 ± 11.09 for the SUD group (Student's *t* = −1.77; *p* = 0.080, ns), while endpoint scores were 23.42 ± 6.85 and 26.94 ± 10.03, respectively (Student's *t* = −1.84; *p* = 0.07, ns), i.e., non-SUD and SUD did not differ on baseline or endpoint scores on the general psychopathology PANSS subscale. However, both groups obtained strong score reductions on this subscale from baseline to endpoint (Student's *t* = 13.11; *p* < 0.00001 for the non-SUD group and Student's *t* = 12.69; *p* < 0.00001 for the SUD).

In the SUD group, VAS craving scores decreased from 7.47 ± 2.45 at baseline to 1.49 ± 2.06 at endpoint (Student's *t* = 12.80; *p* < 0.00001) (Figure 4). The effect size was very large (Cohen's *d* = 2.67; Glass's *delta* = 2.49; Hedges' *g* = 2.67).

We implemented repeated-measures ANOVA involving the independent variable SUD (presence/absence) as the between-subjects variable, time as the within-subjects variable, and CGI, BPRS, PANSS, and VAScrav scores as dependent variables. In the case of statistical significance, we conducted Tukey's *post-hoc* test.

### 3.1 CGI-S

Comparing the 12 timepoints, results indicate a main effect of Time [*F*_(1, 913)_ = 162.798; *p* < 0.0001], with an improvement of clinical global severity.

### 3.2 BPRS

Comparing the 12 timepoints for each subscale of the BPRS, a main effect of time was found for anxiety-depression [*F*_(1, 913)_ = 144.275; *p* < 0.0001], which highlights an overall improvement of symptomatology over time. Moreover, we found an interaction of Time × SUD (presence/absence) [*F*_(1, 913)_ = 4.382; *p* < 0.0001], in which symptoms improve in both conditions over time. For the anergia subscale, there was a main effect of time [*F*_(1, 913)_ = 94.705; *p* < 0.0001], with overall improvement over time. A main effect of time [*F*_(1, 913)_ = 50.333; *p* < 0.0001] was also found for the thought disorder subscale, with an overall improvement of symptoms. Moreover, there was an interaction effect of time × SUD (presence/absence) [*F*_(1, 913)_ = 3.415; *p* < 0.0001], in which symptoms of both conditions improved over time. Main effects of time were also found for the activity [*F*_(1, 913)_ = 53.494; *p* < 0.0001] and hostility/suspiciousness [*F*_(1, 913)_ = 8.443; *p* < 0.0001] subscales and for the BPRS total score [*F*_(1, 913)_ = 105.166; *p* < 0.0001], with overall symptomatologic improvements over time.

### 3.3 PANSS

Comparing the 12 timepoints for each subscale of the PANSS, we found a main effect of time for the positive symptoms subscale [*F*_(1, 913)_ = 35.957; *p* < 0.0001], with an overall improvement in positive symptoms over time. Furthermore, there was an interaction effect of time × SUD (presence/absence) [*F*_(1, 913)_ = 6.493; *p* < 0.0001], in which both conditions improved in positive symptoms over time. A main effect of time was also found for the negative symptoms subscale [*F*_(1, 913)_ = 106.9359; *p* < 0.0001], for the general psychopathology subscale [*F*_(1, 913)_ = 132.715; *p* < 0.0001], and for the PANSS total subscale [*F*_(1, 913)_ = 135.825; *p* < 0.0001], with all symptoms decreasing over time.

### 3.4 VAScrav

Comparing the 12 timepoints for the VAScrav questionnaire in SUD outpatients, we found a main effect of time [*F*_(1, 506)_ = 81.858; *p* < 0.0001], with an overall amelioration in craving over time.

### 3.5 Adverse events

During the study, no serious adverse event developed, but subjective akathisia in a woman led to her drop-out. All adverse events, i.e., nausea, headache, muscle aches, fatigue, and insomnia, were transient and mild, needing no specific treatment or discontinuation. Detailed safety data will be provided in a future study focusing on safety.

## 4 Discussion

In this study, we evaluated 86 patients with schizophrenia, of whom 48 had a comorbid SUD. These patients were all treated with brexpiprazole at the target dose of 4 mg/day; 79 were already treated with other antipsychotics and underwent an appropriate pharmacological switch, whereas seven were antipsychotic drug naïve. We found no differences between the SUD and non-SUD groups, concerning symptoms assessed with the CGI-S, the PANSS, and the 24-item BPRS. We might conclude from our results that people with schizophrenia who have a comorbid SUD do not respond to brexpiprazole treatment less than non-SUD patients with schizophrenia. Hence, having a SUD in a patient with schizophrenia does not confer resistance to treatment with brexpiprazole. Substance craving for their respective substances, as assessed through the VAScrav, was decreased in SUD-comorbid patients during brexpiprazole treatment.

We intended to evaluate the efficacy of treatment with brexpiprazole in psychotic symptom reduction and its ability to improve the global clinical status. Comparing patients with and without comorbid SUD allowed us to evaluate whether brexpiprazole could be a good treatment option in patients with SUD. Comorbid SUD is usually an obstacle to the treatment since it reduces treatment adherence in schizophrenia (86). Furthermore, high-potency dopaminergic blockade in persons with schizophrenia and comorbid SUD may interfere with the reward circuitry (87) and cause dysphoria (88). Lower reward perception may prompt the patient to resume illicit drug use to reinstate their previous state (57, 89). While the activation of D_2_ receptors in the nucleus accumbens soothes the symptoms of opiate withdrawal in opiate-dependent rats, their blockade elicits somatic symptoms attributable to withdrawal (90). The withdrawal symptom-eliciting and aversive effects of dopamine receptor blockade were related to the blockade of D_2_ but not D_1_ dopamine receptors (91). We may presume that by reducing ventral striatal-accumbal dopamine-related reward through D_2_ receptor inhibition, we may actually worsen addictive behaviors in patients with comorbid schizophrenia and SUD, although differences in baseline reward circuitry function among patients with schizophrenia may play a role (92). We did not observe such worsening or lack of improvement in patients receiving brexpiprazole in our study; we may attribute this effect to the partial agonist effect of brexpiprazole on dopamine D_2_/D_3_ receptors in the limbic system (93), mainly to the D_2_ (94). In this study, we showed a positive effect of brexpiprazole 4 mg/day on craving; currently, there are no studies investigating craving in SUD in patients treated with brexpiprazole. One that investigated it in patients with cocaine use disorder found a medium-to-large effect for olanzapine, with Cohen's *d* = 0.79 (95), while we found a much larger effect (Cohen's *d* = 2.67); however, the substance use disorder in their sample differed from ours, as did the craving assessment scale, and sample sizes were different [smaller in Smelson et al. (95)].

We evaluated the psychopathology in our patients using psychometric scales at baseline, i.e., before treatment with brexpiprazole, then every week for 2 months, and then every month until the 6th month of evaluation. We observed the main effects of time for both SUD comorbid and non-comorbid samples for scores on the CGI-S, BPRS-24, and PANSS subscales and total scores. A similar main effect of time with an overall improvement in craving was evident in the SUD-comorbid group. While negative PANSS scores decreased for both SUD and non-SUD groups from baseline to endpoint, and the two groups did not differ for baseline scores on the negative subscale, the final scores of the SUD group were higher than the non-SUD scores, indicating that the latter group's negative symptoms had benefitted from brexpiprazole treatment more than what they did in the SUD group. It should be underlined that the majority of our SUD sample had cannabis use disorder; this subgroup in our study did not show lower negative symptoms compared to the non-SUD sample, in contrast to what has been observed in other studies, where people with cannabis use disorder showed less negative symptoms than individuals without cannabis use disorder (96, 97). There is no sufficient data to speculate as to the neurochemical mechanism underpinning the resistance of negative symptoms to the antipsychotic in SUD patients, but we should recall that most of our sample had cannabis use disorder and that cannabinoid mechanisms may underlie negative symptoms in schizophrenia, although in a most complex way (98).

Regarding differences between SUD and non-SUD groups, the latter had scored higher than the former at baseline on the BPRS Anxiety-Depression subscale. Both populations obtained fair reductions of BPRS scores in this very subscale when treated with brexpiprazole over 6 months. At the study endpoint, the differences in the BPRS Anxiety-Depression subscale disappeared, indicating that depression and anxiety in both SUD comorbid and non-SUD groups with schizophrenia benefitted from drug treatment with brexpiprazole. Decreases in the scores of the BPRS anergy subscale and the negative PANSS dimension were observed with treatment over time.

Patients with comorbid SUD scored higher than their non-comorbid non-SUD counterparts on the BPRS-24 thought disorder subscale at baseline. Both subgroups of patients with schizophrenia improved over time with treatment, with endpoint scores not differing between the two groups. SUD patients scored higher than non-SUD on the PANSS Positive subscale at baseline; both groups responded to drug treatment, with positive symptoms improving over time in both SUD and non-SUD groups.

Patients with comorbid SUD scored higher than non-SUD patients on the BPRS hostility subscale. There is evidence that SUD is related to violent behavior (99); substance use patterns in people with addiction may be related to coping styles associated with aggression and hostility (100). In our study, patients' scores on the hostility subscale of the BRPS decreased over time, independently of whether they had SUD comorbidity or not. The scores on the BPRS activity dimension also decreased over time in both populations.

We have been overcautious in our switch from other antipsychotics to brexpiprazole. Probably, the wash-out we practiced per protocol was not actually needed, as brexpiprazole was well tolerated. In future studies, we are set to switch directly, reducing the dose of the other antipsychotics according to its schedule.

Regarding craving for substances, which we investigated only in the SUD-comorbid population, scores decreased over time, despite the abrupt substance discontinuation and the concomitant use of an antipsychotic such as brexpiprazole. It appears that this antipsychotic has no detrimental effect on the patients' reward system. A recent systematic review focused on the effects of various drugs, including antipsychotics, on cocaine craving (101). This review found no consistent effects of antipsychotics on craving (one study showing positive effects of aripiprazole and two showing results similar to placebo, three studies on risperidone, and one on quetiapine showing no significant results, while among five studies on olanzapine, one showed it to be better than haloperidol, one to be worse than haloperidol, and three showed no significant effects). The results obtained here are legitimate further studies of the effects of antipsychotics on substance craving.

Brexpiprazole was shown to be effective in adult schizophrenia, both in the short- and long-term (102, 103) and as an adjunct, also in major depression (104), even if treatment-resistant (105). There is a current trend to use partial D_2/3_ agonists in the so-called “dual” disorders, i.e., a major psychiatric disorder comorbid with a SUD (106, 107), and there is a sound rationale to pursue this way (108). For the moment, long-acting injectable antipsychotics were associated with improvement in dual disorders (65), and among them, aripiprazole, a partial D_2/3_ agonist, holds a preeminent position (109–111). Future studies will establish whether there are differences among the already marketed partial D_2/3_ agonists aripiprazole, brexpiprazole, and cariprazine in the treatment of comorbid major psychiatric disorders and SUDs (and which SUDs). While evidence for a positive effect of aripiprazole on craving has been obtained in controlled studies (109, 112, 113), data on alcohol use disorder were inconsistent (114, 115). For cariprazine, there are only case reports of efficacy in reducing craving (116, 117), whereas, for brexpiprazole, there are still no reports besides the current study; here, we showed a strong effect of brexpiprazole in reducing craving.

### 4.1 Limitations

Our study had several limitations. Our sample size was small and needs to increase to enable us to draw valid conclusions. Furthermore, there were no comparison groups, for example, samples with or without SUD treated with other than brexpiprazole antipsychotics or placebo. Open-label studies may affect results and limit generalisability. The fact that our study used a population referring to a single site could have limited the representability of the sample. Moreover, we could not analyse data according to gender or whether they were drug-naïve or switched from another antipsychotic, but there were many more women in the non-SUD group and many more men in the SUD group, and the drug-naïve subsample was very small compared to the sample that switched from another antipsychotic. The substances used in the SUD comorbid group were often multiple, and there were not sufficient subsample sizes to allow us to differentiate the different substances. However, most patients in the comorbid group were using cannabis, although the relative majority were multisubstance users (Table 1).

## 5 Conclusion

We found brexpiprazole to be a valid treatment option to treat schizophrenia, with or without substance use disorder. Brexpiprazole proved to be effective on psychotic symptoms, both positive and negative. Comorbid substance use disorder did not confer treatment resistance in this study. Brexpiprazole was found to be suitable to treat patients with comorbid SUD and psychotic disorders since it did not increase craving for illicit substances after their abrupt discontinuation (on the contrary, craving decreased during the study in the SUD group). Furthermore, treatment with brexpiprazole was followed by the leveling of initial differences between SUD and non-SUD patients with schizophrenia on those psychopathological dimensions where the two groups differed at baseline. Further studies with larger samples, randomized control designs, and using healthy controls as comparison groups are needed to confirm these encouraging results. Should our data be confirmed by such studies, new clinical perspectives for the use of brexpiprazole (and partial D_2/3_ agonists in general) may appear in the therapeutic horizon of schizophrenia, bipolar disorder, and other major psychiatric disorders comorbid with specific substance use disorders.

## Data availability statement

The raw data supporting the conclusions of this article will be made available by the authors, without undue reservation.

## Ethics statement

The studies involving humans were approved by CE Lazio 2, Rome, Italy; protocol number 331-306-00387. The studies were conducted in accordance with the local legislation and institutional requirements. Written informed consent for participation in this study was provided by the participants' legal guardians/next of kin.

## Author contributions

GL: Conceptualization, Data curation, Formal analysis, Investigation, Methodology, Software, Supervision, Validation, Writing—original draft, Writing—review & editing. GT: Software, Visualization, Writing—original draft. EA: Data curation, Formal analysis, Methodology, Software, Visualization, Writing—original draft. GK: Conceptualization, Data curation, Formal analysis, Investigation, Methodology, Software, Supervision, Validation, Writing—original draft, Writing—review & editing. FP: Data curation, Formal analysis, Investigation, Methodology, Software, Validation, Writing—original draft. VG: Conceptualization, Investigation, Methodology, Validation, Writing—original draft. AD: Conceptualization, Investigation, Methodology, Software, Validation, Writing—original draft. SD: Conceptualization, Formal analysis, Investigation, Methodology, Project administration, Validation, Visualization, Writing—original draft.
