# Supplementary material for: Brexpiprazole in patients with schizophrenia with or without substance use disorder: an observational study
Source: Front Psychiatry. 2023 Dec 4;14:1321233. doi: 10.3389/fpsyt.2023.1321233 (PMC10725927; doi:10.3389/fpsyt.2023.1321233)
Supplement: Supplementary file 1 [file Table_1.pdf]

Suppl. Table 1. Commonalities and differences in the receptor profiles of the three approved partial dopamine agonist antipsychotic drugs.

	Aripiprazole		Brexpiprazole		Cariprazine	
**Site**	**K_D/K_i (nM)**	**Action**	**K_D/K_i (nM)**	**Action**	**K_D/K_i (nM)**	**Action**
SERT	98–1,080	Inhibitor	65% at 10 μM	Inhibitor		
NET	2,090	Inhibitor	0% at 10 μM	Inhibitor		
DAT	3,220	Inhibitor	90% at 10 μM	Inhibitor		
5-HT$_{1A}$	1.7–5.6	Partial agonist	0.12	Partial agonist	2.6	Partial agonist
5-HT$_{1B}$	830	ND	32	ND		
5-HT$_{1D}$	68	ND	0.47	Antagonist		
5-HT$_{1E}$	8,000	ND	1.9	Antagonist		
5-HT$_{2A}$	3.4–35	Antagonist	12–34	Antagonist	18.8	Antagonist
5-HT$_{2B}$	0.11-0.36	Inverse agonist	140	ND	0.58	Antagonist
5-HT$_{2C}$	15–180	Partial agonist	58	Antagonist	134	Inverse agonist
5-HT$_3$	628	ND	3.7	Antagonist		
5-HT$_{5A}$	1,240	ND	0.12	Partial agonist		
5-HT$_6$	214–786	Antagonist	32	ND		
5-HT$_7$	9.6–39	Antagonist	0.47	Antagonist	84.1	Antagonist
α_{1A}	25.9	ND	3.8	Antagonist	155	Antagonist
α_{1B}	34.4	ND	0.17	Antagonist		
α_{1D}			2.6	Antagonist		
α_{2A}	74.3	ND	15	Antagonist		
α_{2B}	102	ND	17	Antagonist		
α_{2C}	37.9	ND	0.59	Antagonist		
β_1	141	ND	59	Antagonist		
β_2	163	ND	67	Antagonist		
β_3			>10,000	ND		
D_1	265–1,170	ND	160	ND		
D_2	1.4	Partial agonist	0.35	Partial agonist		
D_{2L}	0.74-1.2	Partial agonist	0.30	Partial agonist	0.49	Partial agonist
D_{2S}	1.2	Partial agonist			0.69	Partial agonist
D_3	0.8–9.7	Partial agonist	1.1	Partial agonist	0.085	Partial agonist
D_4	44–514	Partial agonist	6.3	ND		
D_5	95–2,590	ND	ND	ND		
H_1	27.9–61	ND	19	Antagonist	23.2	Antagonist
H_2	>10,000	ND	>10,000	ND		
H_3	224	ND	>10,000	ND		
H_4	>10,000	ND				
mACh			52% at 10 μM	ND	>1,000	Antagonist
M_1	6,780	ND	67% at 10 μM	ND		
M_2	3,510	ND	>10,000	ND		
M_3	4,680	ND				
M_4	1,520	ND				
M_5	2,330	ND				
NMDA	4,001	Antagonist				
σ			96% at 10 μM	ND		

Note. The higher the K_i values, the lower the binding affinity; % potency of receptor occupancy at 10 micromolar concentrations increases as values increase. Blank boxes, not tested. *Abbreviations.* D, dopamine receptors; DAT, dopamine transporter; H, histamine receptors; K_i, dissociation constant, inhibitory; M, muscarinic cholinergic receptors; NAT, noradrenaline transporter; ND, not determined; nM, nanomoles; NMDA, *N*-methyl-D-aspartate glutamate receptors; SERT, serotonin transporter; 5-HT, serotonin receptors; α, alpha adrenoceptors; β, beta-adrenoceptors; μM, micromoles; σ, sigma chaperone intracellular receptors (1 and 2 subtypes). Based on Shapiro et al., 2003 [49] for aripiprazole; Maeda et al., 2014 [47,48] for brexpiprazole; Kiss et al., 2010 [50] and Herman et al., 2018 [51] for cariprazine.
